# Exploring the Sources of Bacterial Spoilers in Beefsteaks by Culture-Independent High-Throughput Sequencing

**DOI:** 10.1371/journal.pone.0070222

**Published:** 2013-07-25

**Authors:** Francesca De Filippis, Antonietta La Storia, Francesco Villani, Danilo Ercolini

**Affiliations:** Division of Microbiology, Department of Agricultural Sciences, University of Naples Federico II, Portici, Italy; University Paris South, France

## Abstract

Microbial growth on meat to unacceptable levels contributes significantly to change meat structure, color and flavor and to cause meat spoilage. The types of microorganisms initially present in meat depend on several factors and multiple sources of contamination can be identified. The aims of this study were to evaluate the microbial diversity in beefsteaks before and after aerobic storage at 4°C and to investigate the sources of microbial contamination by examining the microbiota of carcasses wherefrom the steaks originated and of the processing environment where the beef was handled. Carcass, environmental (processing plant) and meat samples were analyzed by culture-independent high-throughput sequencing of 16S rRNA gene amplicons. The microbiota of carcass swabs was very complex, including more than 600 operational taxonomic units (OTUs) belonging to 15 different phyla. A significant association was found between beef microbiota and specific beef cuts (P<0.01) indicating that different cuts of the same carcass can influence the microbial contamination of beef. Despite the initially high complexity of the carcass microbiota, the steaks after aerobic storage at 4°C showed a dramatic decrease in microbial complexity. *Pseudomonas* sp. and *Brochothrix thermosphacta* were the main contaminants, and *Acinetobacter*, *Psychrobacter* and *Enterobacteriaceae* were also found. Comparing the relative abundance of OTUs in the different samples it was shown that abundant OTUs in beefsteaks after storage occurred in the corresponding carcass. However, the abundance of these same OTUs clearly increased in environmental samples taken in the processing plant suggesting that spoilage-associated microbial species originate from carcasses, they are carried to the processing environment where the meat is handled and there they become a resident microbiota. Such microbiota is then further spread on meat when it is handled and it represents the starting microbial association wherefrom the most efficiently growing microbial species take over during storage and can cause spoilage.

## Introduction

Owing to abundance of nutrients and high water activity, fresh meat can be easily colonized by different types of microorganisms. Microbial growth on meat to unacceptable levels contributes significantly to change meat structure, color and flavor and to cause meat spoilage. Such changes in meat will affect its freshness and spoiled meat will be surely unappealing and unsuitable for human consumption [1], [2]. The initial microbial load of meat depends on the physiological status of the animal at slaughter, the spread of contamination in abattoirs and during processing, while temperature and other conditions during distribution and storage can also influence the rate of spoilage [3], [4]. The microbiota potentially causing spoilage during storage of meat in different conditions has been recently reviewed [5]. Members of *Enterobacteriaceae*, lactic acid bacteria, *Pseudomonas* spp. and *Brochothrix thermosphacta* are recognized as the principal players in meat decay and their dominance during spoilage is influenced by storage conditions such as temperature and oxygen availability [3], [4], [5], [6]. Remarkably, not all the members of the initial microbiota contribute to spoilage. Only a fraction of the initial populations will grow and cause spoilage depending on the storage conditions, such microbes are recognized as Ephemeral/Specific Spoilage micro- Organisms-E(S)SO [4]. Therefore, the concept of succession of spoilage-related microbial groups is very important and studies have been performed to investigate how the microbiota develops and changes during meat storage [2], [4], [7], [8], [9], [10], [11]. However, the initial contamination of meat is a key point that can influence the spoilage dynamics during storage. Multiple sources of contamination can be identified. Carcass contamination takes place during slaughtering by the animal endogenous microbiota. This strongly depends on the hygiene practices at the farmhouse, the conditions of animal transport, the level of automation, the decontamination technologies used and the cleaning practices at the abattoir [12], [13], [14], [15], [16]. Carcass contamination can be also environmental, and the microbiota occurring on tools and surfaces can contribute to the initial microbial load on carcasses. Environmental contaminations can also occur during transport. In addition, subsequent handling of meat in the operations of sectioning and portioning can determine further contamination [17], [18], [19]. All the above possible routes will potentially contribute to the initial composition of the microbiota of meat before the storage starts. The spatial distribution of microbial contaminants on meat has been hypothesized to be not necessarily homogeneous [5]. In fact, some operations such as meat manipulation, slicing and transferring in packages can alter the initial meat microbiota and provide additional contamination in the handling points. In addition, it would be of interest to study in depth whether meat contamination originates directly from the carcass, or if a resident microbiota of the meat processing plant can contribute to contamination. In order to understand which microbial species can contribute to spoilage and what their sources are, it would be important to understand the composition of the microbiota of beef before and after spoilage and to match the members of the microbiota with the microbial populations that are found on carcasses or handling tools and surfaces along the meat chain. Recent applications of high-throughput sequencing (HTS) in foods have proved useful for a quantitative in depth assessment of the changes in microbial populations during food production or storage [20], [21].

In this study, we used culture-independent HTS of 16S rRNA gene amplicons to explore the possible sources of beefsteaks microbial contamination along the meat processing line and to investigate the inter- and intra- steak contamination variability due to handling procedures.

## Materials and Methods

### Sampling

**Figure 1:**
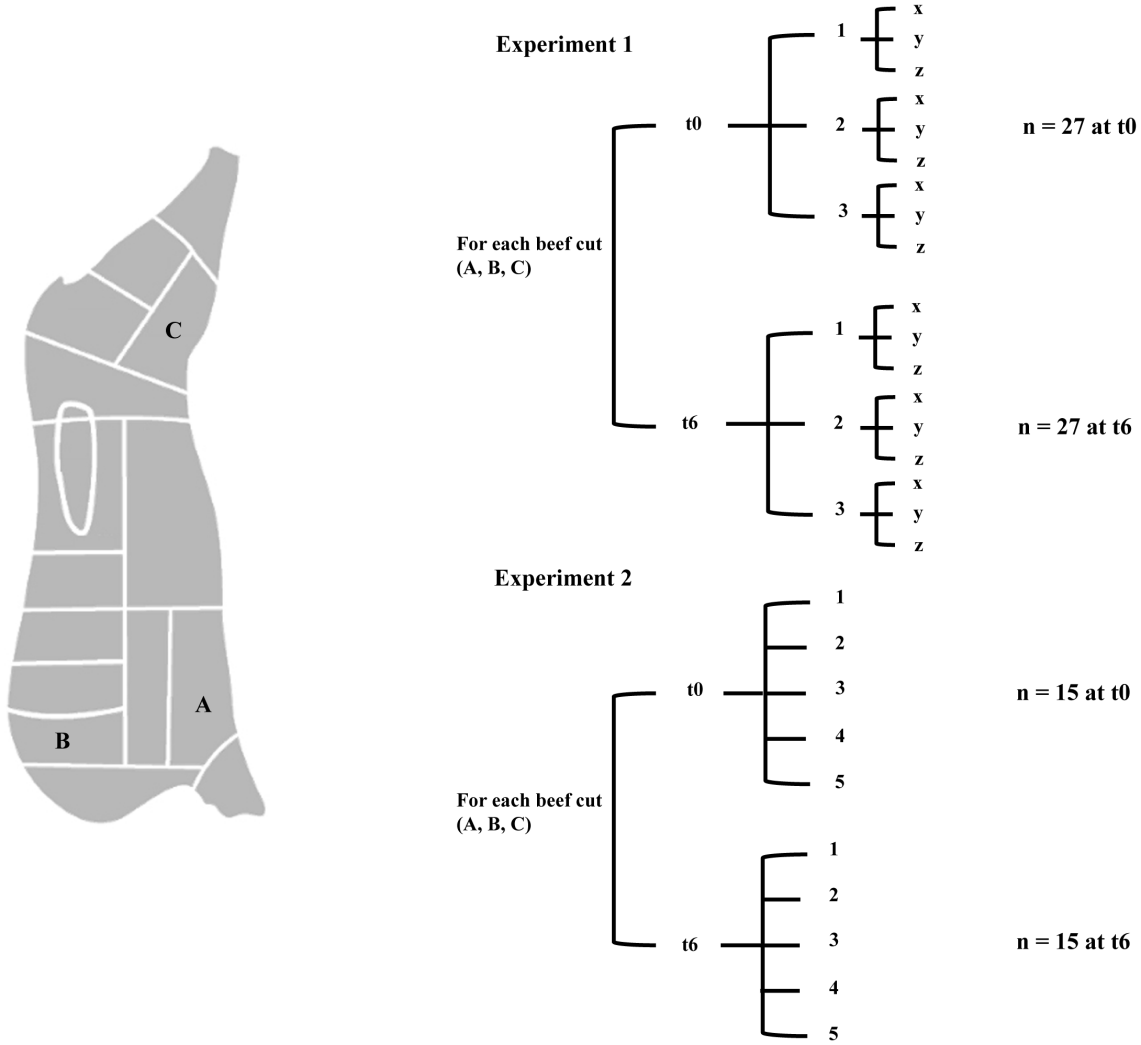
Carcass sampling points used in this study; beef cuts: A, brisket; B, chuck; C, thick-flank. A general scheme of the beefsteaks at time zero (t0) and after one week of aerobic storage at 4°C (t6) analyzed in Experiment 1 and 2 is provided. For Experiment 1, beef steaks were divided in three sub-portions (x, y, z) and analyzed separately.

Two separate carcass samplings in two different slaughterhouses were performed (“Sorrentino s.r.l.” for the first sampling and “Fezza s.n.c.” for the second, both located in Pagani (SA), Italy). A swab sampling of carcasses was performed 12 hours from slaughtering, after washing/before chilling, according to the ISO 17604∶2003 with the modifications indicated in subsequent regulations (Regulations CE 2073/2005 and CE1174/2007). Three sampling points were selected as being the most contaminated (CE 2073/2005) and corresponding to the beef cuts “brisket” (A), “chuck” (B) and “thick-flank” (C) (Figure 1). At each sampling site, a moistened (0.1% buffered peptone water+0.85% sodium chloride solution) sponge (carcass sampling kit, VWR International PBI, Milano Italy) was rubbed vertically, horizontally, and diagonally across the sampling site (100 cm^2^) delineated by a template. The sampled half carcass was followed during the production line and the 3 beef cuts were also sampled at the butchery after portioning (“Macelleria delle rose”, Angri (SA), Italy). The number of samples collected is summarized in Figure 1. In the first sampling (experiment 1), 6 steaks were sampled for each of the 3 beef cuts (n = 18). In addition, swab sampling was performed in the meat processing environment: on the hands of the operator, on the knife used for slicing, on the bench surface where the beef was sectioned (chopping board) and on the cold store wall (n = 4). After collection, all the samples were cooled at 4°C and analyzed within 3 hours. Once transferred to the laboratory, half of the beefsteaks from each muscle were analyzed immediately and the remaining part were singly placed in polystyrene trays and stored aerobically at 4°C for 1 week to achieve spoilage. Prior to microbial analysis and DNA extraction the beefsteaks were divided in 3 portions (x, y and z) that were treated as separate samples (total beef samples n = 27 at time zero and n = 27 after 1 week of storage). The second sampling (experiment 2) was performed in another day in a different slaughterhouse as above described with exception that 5 steaks for each of the 3 muscles were analyzed at time zero (n = 15) and 5 after 1 week of storage at 4°C (n = 15). Moreover, based on the results of the first sampling, the whole steak was analyzed without sub-sectioning.

All the samples were collected and used with the permission of the slaughterhouse and of the butcher.

### DNA Extraction, Amplicon Library Preparation and Sequencing

Total DNA extraction from the meat samples was carried out using the Biostic™ Bacteremia DNA isolation kit (MO BIO Laboratories, Inc. Carlsbad, CA). Steaks were washed in a 5-fold volume of quarter’s strength Ringer solution (Oxoid, Milano, Italy) and a 10 ml aliquot was used for DNA extraction. Ten ml of the 5-fold dilution of beef samples or 20 ml of sponge buffers were centrifuged (12,000 g) and extraction was performed from the pellet resuspended in 1 ml of quarter strength Ringer’s solution.

The microbial diversity was studied by pyrosequencing of the amplified V1–V3 region of the 16S rRNA gene by using primers Gray28F 5′-TTTGATCNTGGCTCAG and Gray519r 5′-GTNTTACNGCGGCKGCTG amplifying a fragment of 520 bp [22]. 454-adaptors were included in the forward primer followed by a 10 bp sample-specific Multiplex Identifier (MID). Each PCR mixture (final volume, 50 µl) contained 50 ng of template DNA, 0.4 µM of each primer, 0.50 mmol l^−1^ of each deoxynucleoside triphosphate, 2.5 mmol l^−1^ MgCl_2_, 5 µl of 10 PCR buffer and 2.5 U of native *Taq* polymerase (Invitrogen, Milano, Italy). The following PCR conditions were used: 94°C for 2 min, 35 cycles of 95°C for 20 s, 56°C for 45 s and 72°C for 5 min, and a final extension at 72°C for 7 min. After agarose gel electrophoresis, PCR products were purified twice by Agencourt AMPure kit (Beckman Coulter, Milano, Italy), quantified using the QuantiFluor™ (Promega, Milano, Italy) and an equimolar pool was obtained prior to further processing. The amplicon pool was used for pyrosequencing on a GS Junior platform (454 Life Sciences, Roche Diagnostics, Italy) according to the manufacturer’s instructions by using a Titanium chemistry.

### Bioinformatics and Data Analysis

Raw reads were first filtered according to the 454 processing pipeline. Sequences were then analyzed and further filtered by using QIIME 1.6.0 software [23]. In order to guarantee a higher level of accuracy in terms of Operational Taxonomic Units (OTUs) detection, after the split library script performed by QIIME, the reads were excluded from the analysis if they had an average quality score lower than 25, if they were shorter than 300 bp and if there were ambiguous base calls. Sequences that passed the quality filter were denoised [24] and singletons were excluded. OTUs defined by a 97% of similarity were picked using the uclust method [25] and the representative sequences were submitted to the RDPII classifier [26] to obtain the taxonomy assignment and the relative abundance of each OTU using the Greengenes 16S rRNA gene database [27].

Alpha diversity was evaluated through QIIME to generate rarefaction curves, Good’s coverage, Chao1 richness [28] and Shannon diversity indices [29]. Beta diversity was evaluated with UniFrac [30]. Weighted UniFrac distance matrices and OTU tables were used to perform Adonis and Anosim statistical tests through the compare_category.py script of QIIME, in order to verify the influence of the time of storage and the different beef cut on the microbial population. ANOVA was performed by StatPlus (5.8.0) from the OTU tables in order to investigate the differences between the sub-portions of beefsteaks analyzed in experiment 1. The OTU taxonomy table and the weighted UniFrac distance matrix generated by QIIME were used to produce heatmaps by using the software TMeV v 4.8 [31].

An OTU network was generated by QIIME and a bipartite graph was constructed in which each node represented either a meat sample or a bacterial OTU. Connections were drawn between samples and OTUs, with edge weights defined as the number of sequences from each OTU that occurred in each sample. Networks were visualized using Cytoscape 2.5.2 [32].

### Nucleotide Sequence Accession Number

All the sequencing data were deposited at the Sequence Read Archive of the National Center for Biotechnology Information (SRP021108).

## Results

In this study, high-throughput sequencing was used to investigate the microbiota of beef from carcasses to beefsteaks after spoilage. Partial 16S rRNA gene sequencing was obtained from DNA directly extracted from environmental swabs as well as beef samples and 16S amplicon pyrosequencing was performed on with a 454 technology. The alpha diversity analysis was performed to investigate the diversity within the samples while the beta diversity analysis was used to assess the diversity between the samples.

### Sequencing and Data Analysis

A total of 562,277 raw sequences were obtained and analyzed; 409,165 reads passed the filters applied through the QIIME split_library.py script, with an average value of 4051 reads/sample and an average length of 457 bp. The number of OTUs, the Good’s estimated sample coverage (ESC), the Chao1 and Shannon indices obtained for all the samples in the two experiments are reported in Table 1 and Table 2, respectively. The rarefaction analysis and the estimated sample coverage indicated that there was satisfactory coverage for all the samples. Carcass and butchery environmental swabs showed a more complex microbiota, as indicated by the Chao1 and Shannon indices. Rarefaction curves showed that at least 3000 reads per sample were necessary to obtain a good coverage for the fresh beefsteaks and the butchery environmental swabs, while more than 4000 reads were necessary for the carcass swabs, which had a more complex microbiota. On the contrary, spoiled beef samples were already covered with about 1000 reads/sample (Figure S1).

**Table 1:** Number of sequences analyzed, observed diversity and estimated sample coverage (Good’s coverage) for 16S rRNA amplicons analyzed in the first experiment.

Sample	Reads	OTUs	Chao1	Shannon	ESC
**A**	6586	801.00	1464.25	7.55	94%
**B**	4909	514.00	900.88	6.36	95%
**C**	5280	375.00	566.34	5.41	97%
**CHOPPING BOARD**	4000	381.00	594.89	5.64	96%
**COLDSTORE**	3436	272.00	494.78	5.12	96%
**HAND**	5019	446.00	783.62	5.62	96%
**KNIFE**	5747	631.00	899.49	6.26	96%
**A1-t0**	1021	333.67	657.26	7.30	77%
**A2-t0**	5139	466.00	672.22	6.37	96%
**A3-t0**	1465	209.00	405.62	5.84	83%
**B1-t0**	4783	432.33	617.99	6.08	97%
**B2-t0**	2281	372.00	629.55	6.22	91%
**B3-t0**	3077	452.33	830.23	6.09	91%
**C1-t0**	4683	338.00	459.80	4.33	97%
**C2-t0**	5315	344.67	476.52	4.27	98%
**C3-t0**	2233	243.33	374.46	4.91	94%
**A1-t6**	2175	77.33	115.70	1.96	98%
**A2-t6**	2421	72.67	107.97	1.46	99%
**A3-t6**	2273	94.67	131.19	1.97	98%
**B1-t6**	2672	168.00	266.68	4.16	97%
**B2-t6**	3029	145.00	190.75	3.10	98%
**B3-t6**	3804	204.67	281.44	4.04	98%
**C1-t6**	3275	140.67	195.52	2.51	98%
**C2-t6**	4469	223.33	287.66	4.19	98%
**C3-t6**	4321	135.33	184.07	2.25	99%

Abbreviations: OTU, operational taxonomic unit; ESC, estimated sample coverage. Chao1, Shannon and ESC were calculated with Qiime at the 3% distance level. Beefsteaks samples at time zero (t0) and after one week of aerobic storage at 4°C (t6) were labeled according to the beef cut of origin: A, brisket; B, chuck; C, thick-flank. The results from the 3 sub-portions of each beefsteak were averaged.

**Table 2:** Number of sequences analyzed, observed diversity and estimated sample coverage (Good’s coverage) for 16S rRNA amplicons analysed in the second experiment.

Sample	Reads	OTUs	Chao1	Shannon	ESC
**A**	5854	930	1567.00	7.53	92%
**B**	7309	883	1481.72	7.49	95%
**C**	6036	747	1270.50	6.57	94%
**CHOPPING BOARD**	3150	204	383.56	3.61	96%
**COLDSTORE**	3329	245	386.27	3.65	97%
**HAND**	6179	220	345.07	3.15	98%
**KNIFE**	6861	252	424.86	3.64	98%
**A1-t0**	1719	377	629.58	6.55	88%
**A2-t0**	2555	236	422.33	3.22	95%
**A3-t0**	2056	291	466.45	4.79	93%
**A4-t0**	5005	712	1184.46	6.66	93%
**A5-t0**	7436	762	1112.86	5.51	96%
**B1-t0**	4516	268	394.29	4.30	97%
**B2-t0**	3669	200	266.98	3.99	98%
**B3-t0**	4331	448	679.60	5.48	96%
**B4-t0**	6902	614	835.35	6.07	97%
**C1-t0**	3980	317	473.88	4.50	97%
**C2-t0**	3634	245	336.12	5.28	98%
**C3-t0**	4828	292	354.34	5.30	99%
**C4-t0**	7955	398	722.01	2.42	97%
**A1-t6**	4384	92	131.67	1.97	99%
**A2-t6**	5447	129	186.40	2.74	99%
**A3-t6**	5682	105	134.53	2.40	99%
**A4-t6**	5069	108	161.81	2.05	99%
**A5-t6**	4232	97	139.50	2.26	99%
**B1-t6**	4171	160	211.00	3.11	99%
**B2-t6**	4781	120	158.33	2.10	99%
**B3-t6**	5193	100	128.96	2.12	99%
**B4-t6**	4100	125	163.33	2.78	99%
**B5-t6**	4893	79	137.58	1.78	99%
**C1-t6**	4778	120	161.00	2.68	99%
**C2-t6**	2336	101	162.88	2.58	98%
**C3-t6**	5071	150	232.50	3.74	99%
**C4-t6**	4315	102	128.25	2.40	99%
**C5-t6**	6054	139	167.28	2.82	99%

Abbreviations: OTU, operational taxonomic unit; ESC, estimated sample coverage. Chao1, Shannon and ESC were calculated with Qiime at the 3% distance level. Beefsteaks samples at time zero (t0) and after one week of aerobic storage at 4°C (t6) were labeled according to the beef cut of origin: A, brisket; B, chuck; C, thick-flank.

### Microbiota Composition of Beefsteaks, Carcass and Environmental Swabs

**Figure 2:**
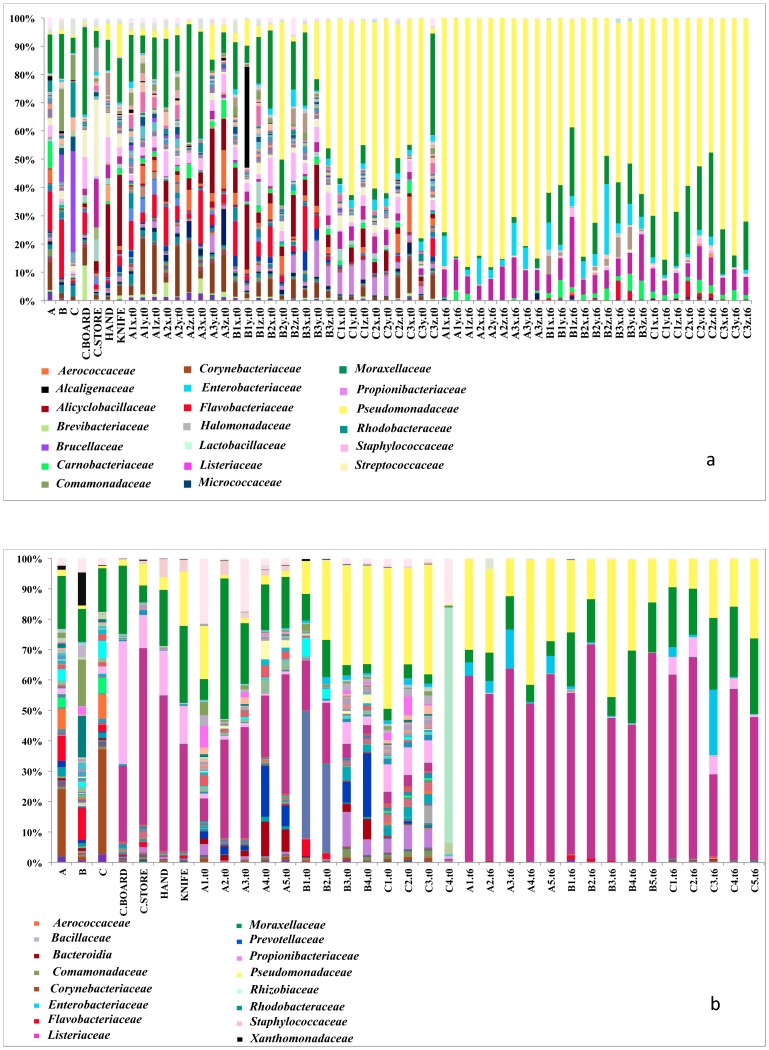
Abundance of bacterial families in carcass swabs, environmental swabs, beefsteaks at time zero (t0) and after one week of aerobic storage at 4°C (t6) in experiment 1 (panel a) and experiment 2 (panel b). Beefsteaks samples are labeled according to the beef cut of origin: A, brisket; B, chuck; C, thick-flank. Color key legend shows only bacterial families with >8% abundance.

The carcass swabs showed a very high degree of microbial diversity; in fact, an average of above 600 OTUs were found in swabs A, B and C, respectively. Fifteen different phyla were present in the carcass swabs, while about 12 were found in the beefsteaks at time zero. However, only 5 were found after one week of storage when the steaks were spoiled. *Bacteroidetes*, *Firmicutes*, *Proteobacteria* and *Actinobacteria* were the most abundant OTUs in the fresh beefsteaks and in the carcass swabs, but only *Firmicutes* and *Proteobacteria* were found in spoiled beef. The complexity of the microbiota of carcass swabs and beefsteaks at time zero is clearly shown in Figure 2. *Moraxellaceae*, *Aerococcaceae*, *Staphylococcaceae*, *Flavobacteriaceae*, *Rhodobacteriaceae* and *Corynebacteriaceae* were the most abundant bacterial families occurring in carcass swabs. These OTUs in different proportions were found in the butchery environmental swabs and in the beefsteaks at time zero along with additional populations. In the case of both slaughterhouses the beefsteaks at time zero from beef cuts A and B were similar between them and had also a microbiota composition similar to the corresponding carcass swabs (Figure 2, panel a and b). However, in both cases the steaks from beef cut C (thick-flank) and the corresponding carcass swab had a different microbial composition. In fact, a predominance of *Pseudomonaceae* was found in all the freshly cut steaks from beef cut C in both sampling experiments (Figure 2, panel a and b). After one week of aerobic storage at 4°C the initial microbiota of beefsteaks changed, showing a significant decrease in microbial diversity (P<0.01). The spoiled steaks after storage were dominated by *Pseudomonaceae*, *Listeriaceae, Moraxellaceae* and *Enterobacteriaceae* in both experiments; in particular, *Pseudomonaceae* and *Listeriaceae* were the dominant groups in experiment 1 and 2, respectively (Figure 2, panel a and b).

The microbial populations in the 3 pieces of the same beefsteak that were analyzed separately (x, y, z) in the experiment 1 were shown to be not significantly different (P>0.05) by ANOVA (Figure 2, panel a). By contrast, in both experiments, the time of storage and the type of beef cut influenced the composition of the bacterial community as measured using Adonis (P<0.01) and Anosim (P<0.005) methods run by QIIME.

**Figure 3:**
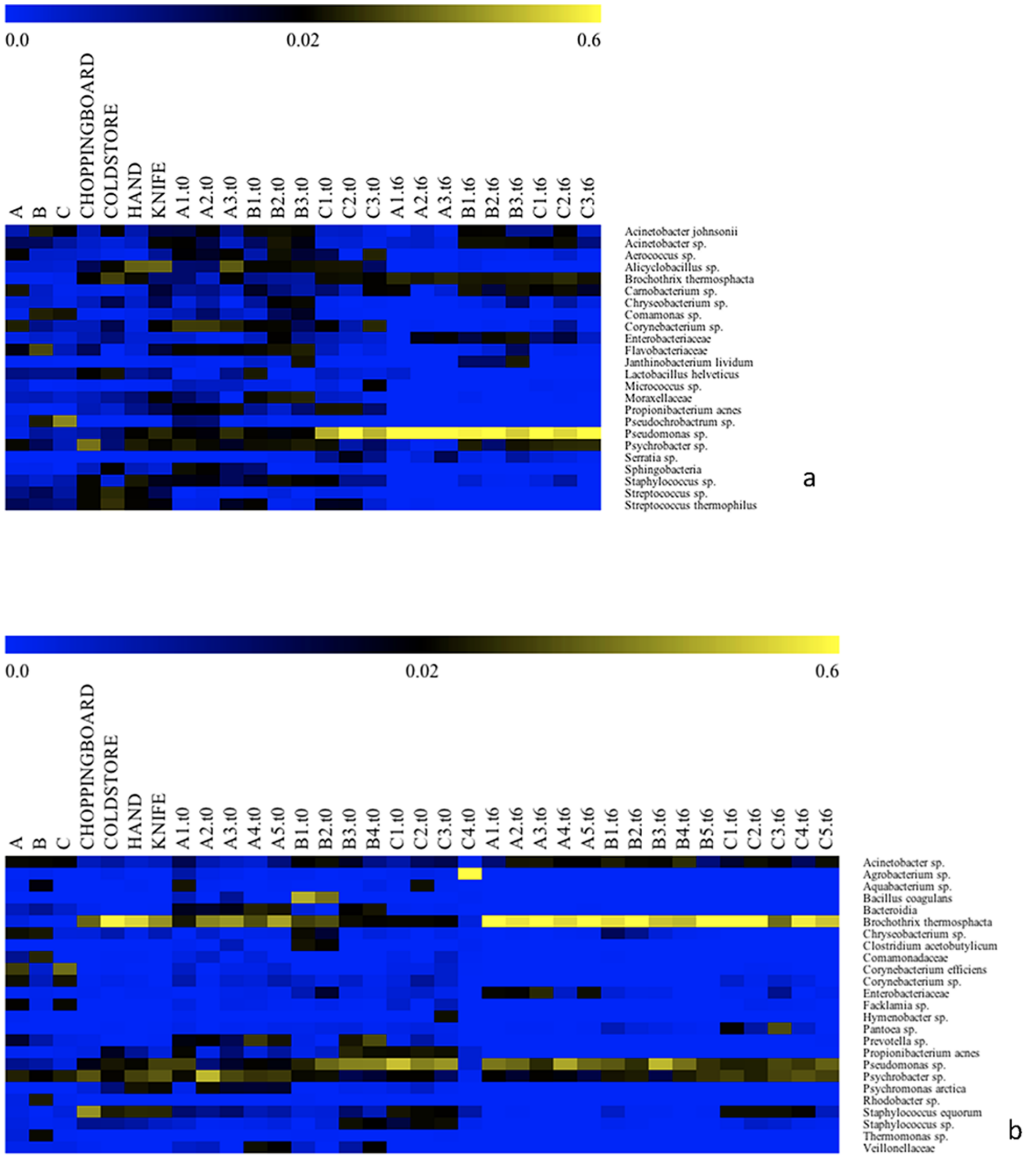
Distribution of bacterial genera and species in carcass swabs, environmental swabs, beefsteaks at time zero (t0) and after one week of aerobic storage at 4°C (t6) in experiment 1 (panel a) and experiment 2 (panel b). Beefsteaks samples are labeled according to the beef cut of origin: A, brisket; B, chuck; C, thick-flank. Only OTUs occurring at>4% abundance in at least 2 samples were included. Abundance of OTUs in the 3 sub-portions of beefsteaks from experiment 1 was averaged. Color scale indicates the relative abundance of each OTU within the samples.

Analyzing the microbial diversity to deeper taxonomic assignment, the succession of genera and species can be observed in the heatmap depicted in Figure 3. In experiment 1, carcass, butchery environmental swabs and beefsteaks at time zero showed a highly complex microbial diversity, while only few species i.e. *Pseudomonas* sp. (always above 50%), *Psychrobacter* sp. and *B. thermosphacta* dominated after 1 week of aerobic storage at 4°C. Additional OTUs in spoiled beefsteaks were *Acinetobacter johnsonii*, *Acinetobacter* sp. and *Carnobacterium* sp. (Figure 3, panel a). Butchery environmental swabs included all the species found in spoiled beef; in addition, lactic acid bacteria, *Alicyclobacillus* sp. and *Staphylococcus* sp. were also abundant (Figure 3, panel a).

In experiment 2 the carcass swabs also had a complex microbiota. However, abundant OTUs such as *Corynebacterium efficiens, Corynebacterium* sp., *Chryseobacterium* sp. and *Facklamia* sp., were not found as dominant bacteria in the corresponding beefsteaks while *Acinetobacter* sp., *Psychrobacter* sp. and *Pseudomonas* sp. were found in both beefsteaks at time zero and after aerobic spoilage. In the butchery environmental swabs, *B. thermosphacta*, *Pseudomonas* sp., *Staph. equorum*, *P. acnes*, *Psychromonas arctica* and *Psychrobacter* sp. were the dominant OTUs. All these species were also the main contaminants of beefsteaks at time zero, while after spoilage the main OTUs found on steaks were *Pseudomonas* sp., *Psychrobacter* sp. and *B. thermosphacta* (Figure 3, panel b). *Enterobacteriaceae* were abundant after storage only in steaks from beef cut A. Remarkably, *P. acnes* found in butchery swabs heavily contaminated the beef at time zero while it was not found after storage. In addition, *Staph. equorum*, which was a main contaminant of butchery environment, was found also in beef at time zero, but it was above 5% abundance only in beefsteaks from beef cut C (Figure 3, panel b). *Acinetobacter* sp. occurred in all the carcass swabs and its abundance ranged between 2 and 12% in spoiled beefsteaks.

### Beta Diversity to Investigate the Contamination Routes

**Figure 4:**
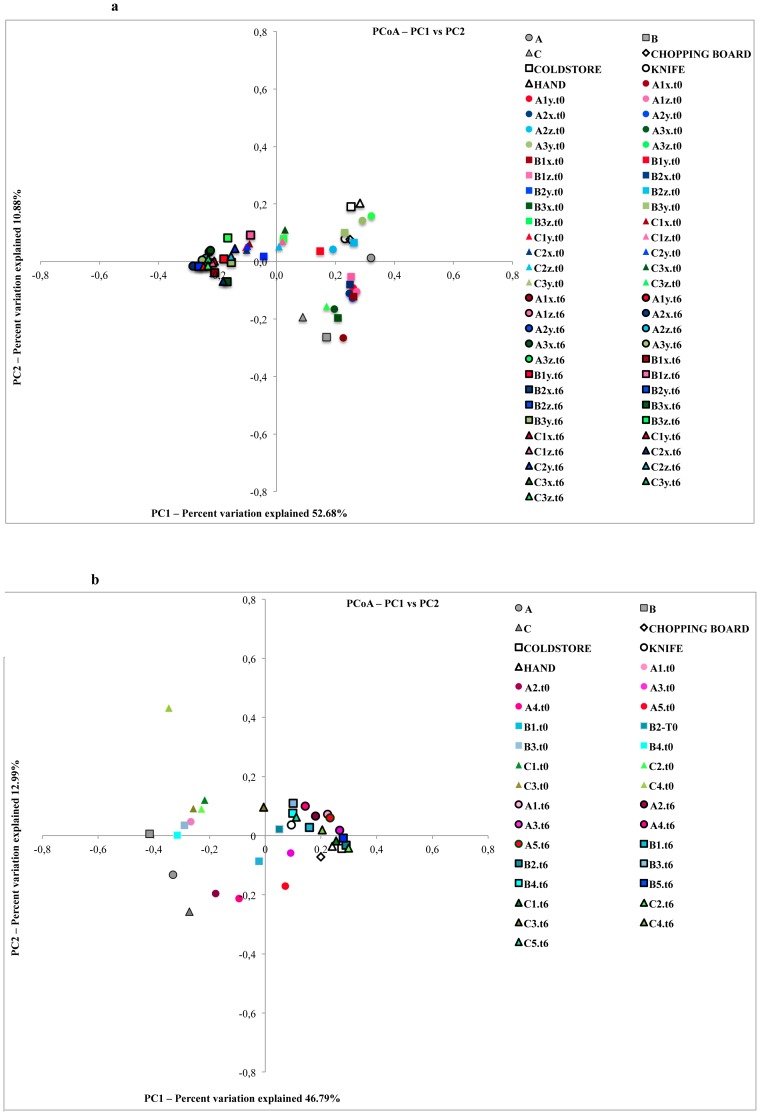
Principal Coordinates Analysis of weighted UniFrac distances for 16S rRNA gene sequence data. Panel a, samples from experiment 1; Panel b, samples from experiment 2. Beef cuts: A, brisket; B, chuck; C, thick-flank. For Experiment 1, beef steaks were divided in three sub-portions (x, y, z) and analyzed separately.

Weighted UniFrac analysis showed that in both experiments fresh and spoiled beefsteaks clustered separately. The carcass swabs clustered with the beefsteaks at time zero in both experiments (Figure 4, panel a and b). However, the butchery environmental swabs clustered with the steaks at time zero in the experiment 1 (Figure 4, panel a) and with the beefsteaks after storage in experiment 2 (Figure 4, panel b). In both cases beefsteaks coming from the same beef cut (labeled with the same symbol) were close in the plot indicating a similar microbial composition (Figure 4). We employed network-based analyses to map meat microbial community composition and structure onto time of storage and sample type (beef cut of origin) thereby complementing the PCoA analyses. Network analyses indicated a clear separation of the OTUs depending on the time of storage and with swabs samples more closely related to microbiota of beefsteaks at time zero; in addition, swabs from the butchery environment had the highest number of shared OTUs highlighting a co-occurring microbial community (Figure S2).

## Discussion

In this study, the microbiota of beefsteaks before and after aerobic storage was analyzed along with carcass swabs of the beef cuts from which the beefsteaks were obtained and swabs of the butchery environment where the beef was handled. Culture-independent HTS was used in order to investigate the changes in composition of bacterial species from carcass to beefsteaks through the production chain and to possibly highlight the sources of contamination of the bacteria grown on beef during storage. For this purpose, two independent experiments were performed in two different slaughterhouses following the samples from carcass to beefsteaks.

Despite the high complexity of the microbiota of the carcass and environmental swabs and of fresh beefsteaks, after aerobic storage at 4°C the bacterial populations showed a dramatic decrease in microbial complexity with only a few dominant species. Therefore, many different microbial species occur in raw meat after animal slaughtering but only very few of them can grow during storage potentially contributing to meat spoilage. This is basically due to competition for the substrate and adaptation to the meat environment, which is better suited for only certain types of bacteria [5], [6], [33]. *Pseudomonas* sp. and *B. thermosphacta* were the main contaminants, representing together more than 80% (75–95%) of the OTUs in the steaks after storage in both experiments. They are both recognized as important aerobic meat spoilers [5]. Different species of *Pseudomonas* have been shown to contribute to meat spoilage especially in aerobic conditions [34]. *P. fragi* has been recognized as the main *Pseudomonas* species associated with beef spoilage [5], [6], [35] and different biotypes of *Ps. fragi* proved capable of determining sensory spoilage of beef by producing spoilage-associated volatile organic compounds during growth in meat at chill temperature [36]. *B. thermosphacta* is facultative anaerobic and it has been shown to be unable to compete against lactic acid bacteria in chill-stored meat under anaerobic conditions [37], [38]; however, it efficiently grows in presence of oxygen and can therefore dominate the meat system along with *Pseudomonas* sp. during aerobic chill storage.

Environmental contamination by skin-associated bacteria such as *P. acnes*, *Staph*. *equorum* and *Staphylococcus* sp. have been often found in the beefsteaks at time zero; however, they were likely outcompeted by the dominant spoilage microbiota during storage as they were never abundant in beefsteaks after one week (Figure 3). *Acinetobacter* sp. was always found in beefsteaks after storage, and it was also detected in carcass as well as environmental swabs (Figure 3). This is in agreement with other studies reporting the occurrence of *Acinetobacter* in meat after aerobic storage [39], [40]. However, such occurrence in the meat environment probably needs more attention as it was recently demonstrated that biofilms by *Acinetobacter* sp. in the meat processing plants could enhance the development of foodborne pathogens such as *Escherichia coli* O157:H7 [41].

The spoilage-associated microbiota found in this study in beefsteaks after storage is typical of aerobic chill storage [4], [5]. Indeed, starting from the same contaminating microbes in carcass and environment, different growth dynamics would take place in other storage conditions such as modified atmosphere or vacuum packaging where other microrganisms such as lactic acid bacteria and facultative anaerobes would dominate the beef system as previously shown [8], [9], [10].

The type of beef cut influenced the composition of the microbiota of beefsteaks. In both experiments the steaks from beef cut C (thick-flank) had a different microbial composition compared to those coming from other beef cuts. In fact, a predominance of *Pseudomonas* sp. was found in all the steaks from beef cut C in both sampling experiments (Figure 2). On the contrary, steaks from beef cut A (brisket) and B (chuck) cluster together in both the experiments, owing to their similar and more complex microbiota (Figure 4). Both cuts are from the front part of the half-carcass. This is recognized as the most contaminated part. In fact, the half-carcass after skinning is hung up by the hind legs and beef exudates, along with water used to wash the carcass, flow from the back to the anterior part, moving the contamination in this direction [14], [42], [43]. Lindbald and Berking [44] have recently shown that all the three beef cuts analyzed in this study are characterized by a high level of contamination in cattle carcasses; however, the data provided are only based on total viable and *Enterobacteriaceae* counts and the level of complexity of the microbial diversity of the samples is not taken into account.

With the sampling performed during experiment 1, we also showed that there are no significant differences in the microbiota between different parts of the same steak, suggesting that the same spoilage dynamics can be expected on the same steak and that point specific contamination is not likely.

All the microbial species developing on beefsteaks during storage were present, although with low abundances, on the surface of carcasses. Therefore, the potential spoilers originally come from the carcass. However, on the basis of the evolution of OTUs abundance noticed in this study, it can be speculated that the spoilage bacteria are transferred from abattoir to the butchery environment and there they start to proliferate in microenvironments where meat residues and exudates can act as substrates. The microbes establish in cold rooms and on the surface of tools such as knives or chopping boards and they constitute a resident microbiota. This resident microbiota can be the final source of contamination of beefsteaks once they are prepared and the storage starts. This is clear from the distribution of OTUs in the butchery environmental swabs, where the abundance of OTUs responsible of meat spoilage increases compared to the carcass swabs (e.g. the case of *B. thermosphacta*, *Pseudomonas* sp. and *Psychrobacter* sp. shown in Fig. 3). As further evidence, the environmental swabs are in some cases close to the beefsteaks after storage as shown by UniFrac and OTUs network analyses (Fig. 4B and Fig. S2).

### Conclusion

The beefsteaks microbiota after storage is selected from a wide initial microbial complexity by meat environment, storage conditions and microbial competition. The spoilage-associated microbial species originate from carcasses, they are carried to the butchery environment where the meat is handled and there they become a resident microbiota. Such microbiota is then further spread on meat when it is sliced or chopped and it represents the starting microbial association wherefrom the most efficiently growing microbial species take over during storage and cause spoilage because of the metabolic activities carried out in beef. On the basis of such evidence utmost care in hygienic practices is needed not only in the handling of carcasses at abattoirs but also in the cleaning of tools and surfaces that will be in contact with meat in order to reduce the types and amounts of bacteria that can contaminate the meat and cause spoilage.

## Supporting Information

Figure S1 **Rarefaction curves obtained by QIIME for representative swabs and meat samples from the experiment 2.**
(TIF)

Figure S2 **Simplified illustration of possible meat-microbe networks.** Network diagrams are color coded by beef cut, type of sample and time of storage. Only OTUs with abundance>0.1% were considered. Panel a, experiment 1; Panel b, experiment 2.
(TIFF)

## References

[1] Fung DY (2010) Microbial Hazards in food: food-borne infections and intoxications. In: F. Toldrà, editor, Handbook of meat processing. Blackwell Publishing. 481–500.

[2] Gram L, Ravn L, Rasch M, Bruhn JB, Christensen AB, et al (2002) Food spoilage-interactions between food spoilage bacteria. Int J Food Microbiol 78: 79–97. 12222639 10.1016/s0168-1605(02)00233-7

[3] Nychas GJE, Marshall DL, Sofos JN (2007) Meat, poultry, and seafood. In Doyle MP, Beuchat LR, editors. Food microbiology: fundamentals and frontiers. Washington: ASM Press. 105–140.

[4] Nychas GJE, Skandamis PN, Tassou CC, Koutsoumanis KP (2008) Meat spoilage during distribution. Meat Sci 78: 77–89. 22062098 10.1016/j.meatsci.2007.06.020

[5] Doulgeraki AI, Ercolini D, Villani F, Nychas GJE (2012) Spoilage microbiota associated to the storage of raw meat in different conditions. Int J Food Microbiol 157: 130–141. 22682877 10.1016/j.ijfoodmicro.2012.05.020

[6] Labadie J (1999) Consequences of packaging on bacterial growth. Meat is an ecological niche. Meat Sci 52: 299–305. 22062579 10.1016/s0309-1740(99)00006-6

[7] Chenoll E, Macian MC, Elizaquivel P, Aznar R (2007) Lactic acid bacteria associated with vacuum packed cooked meat product spoilage: population analysis by rDNA based methods. J Appl Microbiol 102: 498–508. 17241356 10.1111/j.1365-2672.2006.03081.x

[8] Ercolini D, Russo F, Torrieri E, Masi P, et al (2006) Changes in the spoilage-related microbiota of beef during refrigerated storage under different packaging conditions. Appl Environ Microbiol 72: 4663–4671. 16820458 10.1128/AEM.00468-06 PMC1489361

[9] Ercolini D, Ferrocino I, La Storia A, Mauriello G, Gigli S, et al (2010) Effect of a nisin-activated antimicrobial packaging on spoilage-related microbial populations during chilled storage of vacuum-packed beef. Food Microbiol 27: 137–143. 19913704 10.1016/j.fm.2009.09.006

[10] Ercolini D, Ferrocino I, Nasi A, Ndagijimana M, Vernocchi P, et al (2011) Monitoring of microbial metabolites and bacterial diversity in beef stored in different packaging conditions. Appl Environ Microbiol 77: 7372–7381. 21803905 10.1128/AEM.05521-11 PMC3194879

[11] Pennacchia C, Ercolini D, Villani F (2011) Spoilage-related microbiota associated with chilled beef stored in air or vacuum pack. Food Microbiol 28: 84–93. 21056779 10.1016/j.fm.2010.08.010

[12] Galland JC (1997) Risks and prevention of contamination of beef carcasses during the slaughter process in the United States of America. Rev Sci Tech OIE 16: 395–404. 10.20506/rst.16.2.1023 9501353

[13] Sheridan JJ (1998) Sources of contamination during slaughter and measures for control. J Food Safety 18: 321–339.

[14] Yalcin S, Nizamlioclu M, Gurbuz U (2001) Fecal coliform contamination of beef carcasses during the slaughtering process. J Food Safety 21: 225–231.

[15] Steele FM, McMullin DQ (2007) The examination of surface contamination on beef carcasses during slaughter and aging in a small-scale meat packaging operation equipped with an organic acid carcass washer. J Anim Vet Adv 6: 927–931.

[16] Abdalla MA, Suliman SE, Bakhiet AO (2010) Method for reducing contamination of indigenous cattle carcasses during slaughtering. Assiut Vet Me J 56: 86–93.

[17] Bjorkroth KJ, Korkeala HJ (1997) Use of rRNA gene restriction patterns to evaluate lactic acid bacterium contamination of vacuum-packaged sliced cooked whole-meat product in a meat processing plant. Appl Environ Microbiol 63: 448–453. 9023922 10.1128/aem.63.2.448-453.1997 PMC168334

[18] Aslam M, Greer GG, Nattress FM, Gill CO, McMullen LM (2004) Genotypic analysis of *Escherichia coli* recovered from product and equipment at a beef-packing plant. J Appl Microbiol 97: 78–86. 15186444 10.1111/j.1365-2672.2004.02277.x

[19] Vihavainen E, Lundstrom HS, Susiluoto T, Koort J, Paulin L, et al (2007) Role of broiler carcasses and processing plant air in contamination of modified-atmosphere-packaged broiler products with psychrotrophic lactic acid bacteria. App Environ Microb 73: 1136–1145. 10.1128/AEM.01644-06 PMC1828681 17142357

[20] Ercolini D (2013) High-throughput sequencing and metagenomics: moving forward in the culture-independent analysis of food microbial ecology. Appl Environ Microbiol 79: 3148–3155. 23475615 10.1128/AEM.00256-13 PMC3685257

[21] Bokulich NA, Mills DA (2012) Next-generation approaches to the microbial ecology of food fermentations. BMB Rep 45: 377–389. 22831972 10.5483/bmbrep.2012.45.7.148

[22] Ercolini D, De Filippis F, La Storia A, Iacono M (2012) “Remake” by high-throughput sequencing of the microbiota involved in the production of water buffalo mozzarella cheese. Appl Environ Microbiol 78: 8142–8145. 22941080 10.1128/AEM.02218-12 PMC3485941

[23] Caporaso JG, Kuczynski J, Stombaugh J, Bittinger K, Bushman FD, et al (2010) QIIME allows analysis of high-throughput community sequencing data. Nat Methods 7: 335–336. 20383131 10.1038/nmeth.f.303 PMC3156573

[24] Reeder J, Knight R (2010) Rapidly denoising pyrosequencing amplicon reads by exploiting rank-abundance distributions. Nat Methods 7: 668–669. 20805793 10.1038/nmeth0910-668b PMC2945879

[25] Edgar RC (2010) Search and clustering orders of magnitude faster than BLAST. Bioinformatics 26: 2460–2461. 20709691 10.1093/bioinformatics/btq461

[26] Wang Q, Garrity GM, Tiedje JM, Cole JR (2007) Naïve Bayesan classifier for rapid assignment of rRNA sequences into the new bacterial taxonomy. Appl Environ Microbiol 73: 5261–5267. 17586664 10.1128/AEM.00062-07 PMC1950982

[27] McDonald D, Price MN, Goodrich J, Nawrocki EP, De Santis TZ, et al (2012) An improved Greengenes taxonomy with explicit ranks for ecological and evolutionary analyses of bacteria and archea. ISME J 6: 610–618. 22134646 10.1038/ismej.2011.139 PMC3280142

[28] Chao A, Bunge J (2002) Estimating the number of species in a stochastic abundance model. Biometrics 58: 531–539. 12229987 10.1111/j.0006-341x.2002.00531.x

[29] Shannon CE, Weaver W (1949) The mathematical theory of communication. Urbana: University of Illinois Press. 125 p.

[30] Lozupone C, Knight R (2005) UniFrac: a new phylogenetic method for comparing microbial communities. Appl Environ Microbiol 71: 8228–8235. 16332807 10.1128/AEM.71.12.8228-8235.2005 PMC1317376

[31] Saeed AI, Sharov V, White J, Li J, Liang W, et al (2003) TM4: a free, open-source system for microarray data management and analysis. Biotechniques 34: 374–378. 12613259 10.2144/03342mt01

[32] Shannon P, Markiel A, Ozier O, Baliga NS, Wang JT, et al (2003) Cytoscape: a software environment for integrated models of biomolecular interaction networks. Genome Res 13: 2498–2504. 14597658 10.1101/gr.1239303 PMC403769

[33] Cornu M, Billoir E, Bergis H, Beaufort A, Zuliani V (2011) Modeling microbial competition in food: Application to the behavior of *Listeria monocytogenes* and lactic acid flora in pork meat products. Food Microbiol 28: 639–647. 21511123 10.1016/j.fm.2010.08.007

[34] Liao CH (2006) Pseudomonas and related genera. In: C. W. Blackburn, editor, Food spoilage microorganisms. Cambridge: Woodhead Publishing Limited. 213–286.

[35] Ercolini D, Russo F, Blaiotta G, Pepe O, Mauriello G, et al (2007) Simultaneous detection of *Pseudomonas fragi*, *P. lundensis* and *P. putida* from meat by a multiplex PCR assay targeting the *carA* gene. Appl Environ Microbiol 73: 2354–2359. 17293505 10.1128/AEM.02603-06 PMC1855653

[36] Ercolini D, Casaburi A, Nasi A, Ferrocino I, Di Monaco R, et al (2010) Different molecular types of *Pseudomonas fragi* have the same overall behaviour as meat spoilers. Int J Food Microbiol 142: 120–131. 20627208 10.1016/j.ijfoodmicro.2010.06.012

[37] Russo F, Ercolini D, Mauriello G, Villani F (2006) Behaviour of *Brochothrix thermosphacta* in presence of other meat spoilage microbial groups. Food Microbiol 23: 797–802. 16943085 10.1016/j.fm.2006.02.004

[38] Sakala RM, Hayashidani H, Kato Y, Hirata T, Makino Y, et al (2002) Change in the composition of the microflora on vacuum-packaged beef during chiller storage. Int J Food Microbiol 74: 87–99. 11929174 10.1016/s0168-1605(01)00732-2

[39] Buys EM, Nortje GL, Jooste PJ, Von Holy A (2000) Bacterial populations associated with bulk packaged beef supplemented with dietary vitamin E. Int J Food Microbiol. 56: 239–244. 10.1016/s0168-1605(00)00158-6 10857551

[40] Olsson C, Ahrne S, Pettersson B, Molin G (2003) The bacterial flora of fresh and chill-stored pork: analysis by cloning and sequencing of the 16S rRNA genes. Int J Food Microbiol 83: 245–252. 12745230 10.1016/s0168-1605(02)00372-0

[41] Habimana O, Heir E, Langsrud S, Asli AW, Møretrø T (2010) Enhanced surface colonization by Escherichia coli O157:H7 in biofilms formed by an *Acinetobacter calcoaceticus* isolate from meat-processing environments. Appl Environ Microbiol 76: 4557–4559. 20453142 10.1128/AEM.02707-09 PMC2897464

[42] Bell RG (1997) Distribution and sources of microbial contamination on beef carcasses. J Appl Microbiol 82: 292–300. 12455892 10.1046/j.1365-2672.1997.00356.x

[43] Eisel WG, Linton RH, Muriana PM (1997) A survey of microbial levels for incoming raw beef, environmental sources, and ground beef in a red meat processing plant. Food Microbiol 14: 273–282.

[44] Lindbald M, Berking C (2013) A meat control system achieving significant reduction of visible faecal and ingesta contamination of cattle, lamb and swine carcasses at Swedish slaughterhouses. Food Control 30: 101–105.

