# Supplementary figures and images for: Exploring the Sources of Bacterial Spoilers in Beefsteaks by Culture-Independent High-Throughput Sequencing
Source: PLoS One. 2013 Jul 25;8(7):e70222. doi: 10.1371/journal.pone.0070222 (PMC3723795; doi:10.1371/journal.pone.0070222)

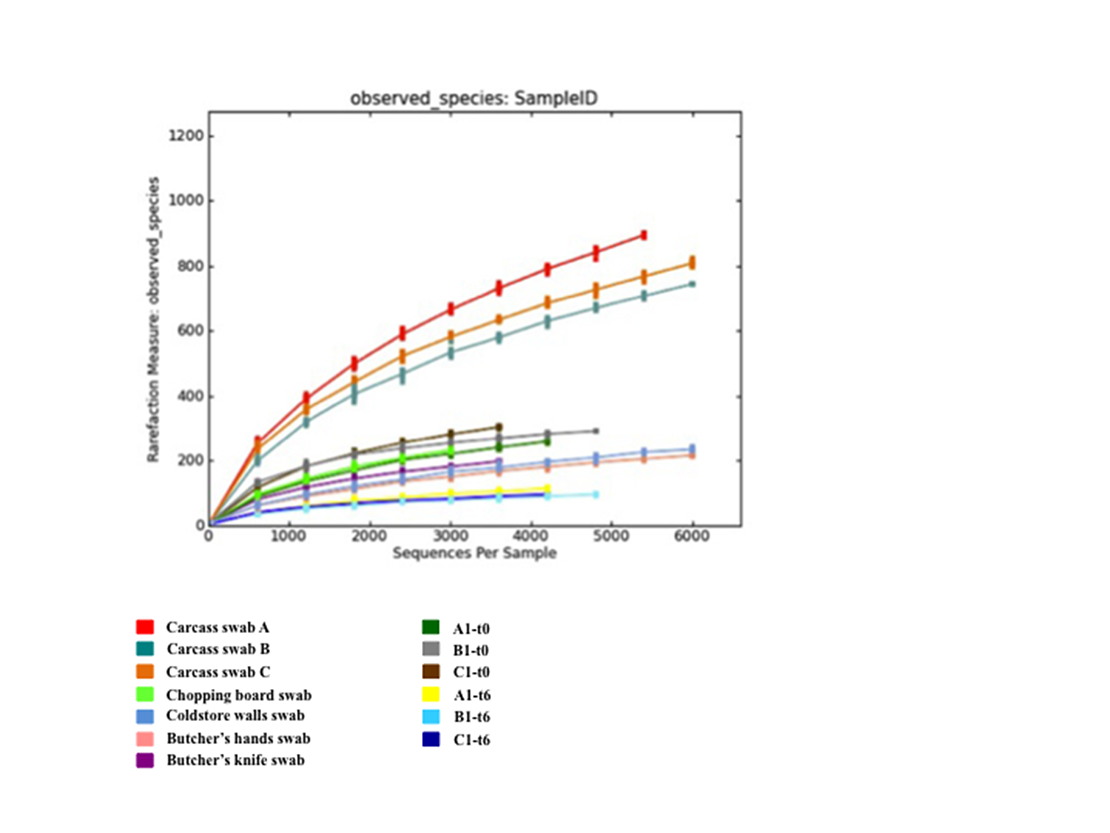

Supplement: Figure S1 — Rarefaction curves obtained by QIIME for representative swabs and meat samples from the experiment 2. (TIF) [file pone.0070222.s001.tif]

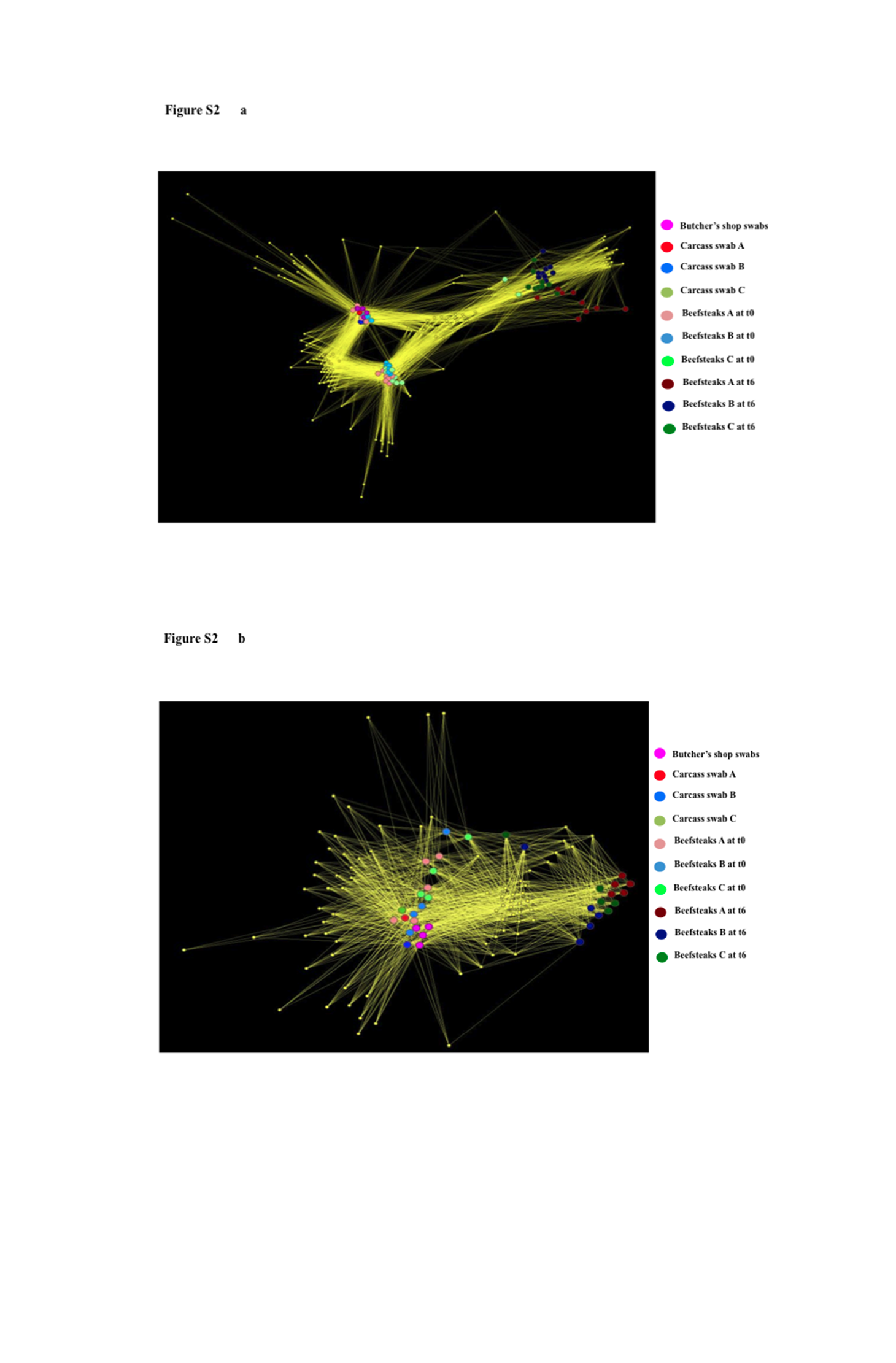

Supplement: Figure S2 — Simplified illustration of possible meat-microbe networks. Network diagrams are color coded by beef cut, type of sample and time of storage. Only OTUs with abundance>0.1% were considered. Panel a, experiment 1; Panel b, experiment 2. (TIFF) [file pone.0070222.s002.tif]
